# Microarray Meta-Analysis of RNA-Binding Protein Functions in Alternative Polyadenylation

**DOI:** 10.1371/journal.pone.0090774

**Published:** 2014-03-12

**Authors:** Wenchao Hu, Yuting Liu, Jun Yan

**Affiliations:** CAS-MPG Partner Institute for Computational Biology, Shanghai Institutes for Biological Sciences, Chinese Academy of Sciences, Shanghai, China; The John Curtin School of Medical Research, Australia

## Abstract

Alternative polyadenylation (APA) is a post-transcriptional mechanism to generate diverse mRNA transcripts with different 3′UTRs from the same gene. In this study, we systematically searched for the APA events with differential expression in public mouse microarray data. Hundreds of genes with over-represented differential APA events and the corresponding experiments were identified. We further revealed that global APA differential expression occurred prevalently in tissues such as brain comparing to peripheral tissues, and biological processes such as development, differentiation and immune responses. Interestingly, we also observed widespread differential APA events in RNA-binding protein (RBP) genes such as *Rbm3*, *Eif4e2* and *Elavl1*. Given the fact that RBPs are considered as the main regulators of differential APA expression, we constructed a co-expression network between APAs and RBPs using the microarray data. Further incorporation of CLIP-seq data of selected RBPs showed that Nova2 represses and Mbnl1 promotes the polyadenylation of closest poly(A) sites respectively. Altogether, our study is the first microarray meta-analysis in a mammal on the regulation of APA by RBPs that integrated massive mRNA expression data under a wide-range of biological conditions. Finally, we present our results as a comprehensive resource in an online website for the research community.

## Introduction

In the last step of eukaryotic transcription, the mRNA transcripts are cleaved at the 3′ termini of the transcripts at so-called poly(A) site. Then poly(A) tails of 100–200 nt are added in a process named polyadenylation. This is a tightly regulated process involving the interplay between 3′UTRs that contain different cis-regulatory elements and RNA Binding Proteins (RBPs). RBPs such as cytoplasmic polyadenylation specific factor (CPSF) and cleavage stimulation factor (CstF) can recognize and bind to specific sequence motifs upstream and downstream of poly(A) site to enable the cleavage on the specific poly(A) site. Typically one or more alternative poly(A) sites exist for mammalian genes, resulting transcripts with different 3′UTRs for the same gene, in an event called alternative polyadenylation (APA). It can even produce new proteins with novel C-termini or lead to non-sense mediated decay (NMD) when acting upstream of a stop codon [1]–[3]. Similar to alternative initiation and alternative splicing, APA is capable to generate transcript diversity and has been found increasingly important in gene regulation.

RBP and miRNA are known to interact with mRNA transcripts in their 3′UTRs. Transcript isoforms resulting from APA may have different expression levels due to different sets of RBP or miRNA binding sites in their 3′ UTRs. Although some poly(A) sites are more constitutively expressed than the others on the same gene, it has also been shown that the choices of poly(A) sites are tissue-specific and can change under different biological conditions. Zhang et al. examined possible poly(A) sites from existing mRNA and EST databases and found that the transcripts using different poly(A) sites express in a tissue specific manner [4]. Recent study using next generation sequencing technology targeting poly(A) site (polyA-seq) also showed that many tissue-specific poly(A) site usages are conserved across species [5]. Furthermore, Sanberg et al. (2008) and Ji et al. (2009) demonstrated that global poly(A) site usage may shift in cancer and during cell proliferation. In particular, they found that cells during proliferation tend to prefer the isoforms with shorter 3′ UTRs [6], [7]. They hypothesized that this mechanism may help the cells to escape the inhibitory effect from miRNA targeting the longer 3′ UTRs and therefore enable the up-regulation of genes during proliferation. During the process of development, it was also found that zebrafish and mouse tend to express the transcripts with lengthened 3′ UTRs [8]–[10].

RBPs have increasingly been recognized as important regulators of RNA metabolism at the post-transcriptional level. A large number of RBPs are known to bind the 3′UTRs of mRNA transcripts affecting their expression, stability and/or translation. In particular, RBPs play important role in APA and alternative splicing regulation. Furthermore, APA and alternative splicing are frequently coupled together and share common RBP regulators, such as U1 snRNP [11], CELF1 [12], Nova2 [13], and SRSF1 [14]. Benefited from the rapid development of the next-generation deep sequencing technology, several RBPs regulating APAs have been intensively studied and the underlying mechanisms have been proposed. For example, PABPN1 has been shown to bind and repress the usage of proximal weak poly(A) sites in APA selection [15]. CPEB1 mediates the shortening of hundreds of 3′ UTRs by recruiting poly(A) machinery to enhance the usage of proximal poly(A) sites during proliferation and tumorigenesis [16]. ELAV binds to proximal poly(A) sites and suppresses their usage for several hundreds of genes and has been supposed to contribute to neuron specific 3′ UTR lengthening in *Drosophila* [17]. PTB can bind to the downstream element (DSE) of poly(A) sites and compete with CstF to inhibit cleavage [18]. Interestingly, many of RBPs were found to be auto-regulated such as ELAV [19], SRSF1 [14], and Tardbp [20]. However, the specific mechanisms underlying RBP-APA regulation have not yet been fully revealed.

Previously, we observed that two APA isoforms of an RBP, RNA-binding protein motif 3 (*Rbm3*), showed opposite expression changes during hypothermia and sleep-deprivation in mouse [21]. The mechanism underlying the differential expression of *Rbm3* APA is still unclear. In this study, we hoped to search for the existence of more such differential expression among APA isoforms systematically in public microarray database. We identified new candidates including *Eif4e2*, *Cd38*, and *Elavl1* in addition to *Rbm3* that contain APAs with differential responses under a wide range of biological conditions. Furthermore, we found that global shift of differential APA expression is dependent on tissue or cell type, disease, and developmental states. Focusing on differential APA expression regulated by RBPs, we developed a co-expression analysis to predict the novel functions of RBPs in APA regulation. Finally, we demonstrated how to infer the functional link between APA differential expression and RBP regulation by incorporating the result of co-expression analysis and RNA binding data of selected RBPs.

## Materials and Methods

### Construction of alternative 3′ UTR database

The Known Gene (KG) annotations of mouse genome (Mm9) were extracted from UCSC genome browser. The information of 3′ ends of mRNA transcripts was integrated into poly(A) site database. We extracted 3′ UTRs from KG annotations and merged them into clusters if any overlaps occurred.

Polya_DB2 is a comprehensive poly(A) database across different animals constructed by Lee et al. [22]. We downloaded all poly(A) sites in Polya_DB2 for mouse Mm5 genome. The genomic coordinates in Mm5 were converted to Mm9 genome using liftOver program [23].

Data from a recent study using polyA-seq generated poly(A) site atlas for 24 tissues from human, rhesus, dog, mouse, and rat was downloaded from UCSC browser. We extracted poly(A) sites for mouse and merged them into clusters if the adjacent poly(A) sites are within the distance of 40 bps. This dataset gave rise to 126,028 poly(A) site clusters in total. We further required that one poly(A) cluster should be represented in at least two tissues and thus reduced the number of poly(A) site clusters to 39,710. Finally, we merged poly(A) sites from this dataset with the 3′ ends of KG genes and polyA_DB2 into the final poly(A) site database. The poly(A) sites within 40 bps were merged and the poly(A) clusters at the 3′-most sites were retained.

We assigned each poly(A) site to the associated 3′ UTR cluster if it falls within the 3′UTR or at most 3,000 bps downstream. In the latter case, the 3′ UTR was also extended downstream to the poly(A) site. Then we defined sub-3′ UTR regions as the regions on 3′ UTRs partitioned by poly(A) sites. The pairs of sub-3′ UTRs from the same host gene were considered as potential APA events. We classified the type of APA event as either tandem UTR if its sub-UTRs are located on the same 3′ UTR or alternative UTRs if located on different exons. Thus we defined 42,735 tandem UTR and 17,756 alternative UTR events for a total of 12,250 mouse genes.

### Customized annotation of Affymetrix Mouse4302 platform for APA analysis

To enable APA expression analysis using microarray data, we mapped all Affymetrix probes from Mouse4302 microarray onto Mm9 mouse genome by Bowtie software [24]. We kept the mappings that are perfect match and unique on both 3′ UTR and KG gene exon regions. We only selected the sub-UTRs targeted by at least three probes. The probes were then grouped into our customized probesets. Finally, we were able to associate 6,220 tandem UTR and 1,859 alternative UTR events for a total of 4,364 genes with our customized probesets on the microarray.

For the mock tandem UTR control, the remaining probes of Affymetrix probesets were equally divided into two adjacent genomic regions each containing at least four probes. Thus, we obtained 7,181 mock tandem UTR control events.

### Quantification of differential APA expression

To calculate differential expression of a specific APA event, we defined UTR Lengthening Index (*ULI*) for tandem UTR events by subtracting expression change of common UTR (*com*) from that of extended UTR (*ext*):
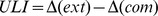



Furthermore, for every microarray experiment we calculated Pearson's correlation *r* for the pair of UTRs of every APA event. Likewise, we calculated correlation *r′* for mock tandem UTRs. The statistical distribution of *r′* can be used as a background distribution to reflect the situation when APA machinery is not perturbed. Therefore we normalized *r* according to its position in the distribution of *r′* (mock background) as *B* value, i.e.
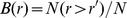
where 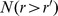 is the number of *r′* less than *r* and *N = 7,181* is the total number of *r′*. In this case, the *B* value represents how significant the differential expression of APA is compared to random background. Similar to this, we have also normalized other statistical quantities such as Two-way ANOVA *p* values for APA events from their mock background distributions.

### Microarray data analysis

All 1,365 mouse4302 microarray series (experiments) available until Sep. 2011 from Gene Expression Omnibus (GEO) were retrieved in the format of raw CEL files. The complete list of experiments can be found in Table S3. We removed the super series and those series with less than four samples for further investigation. The textual annotations for the biological description of each sample on the microarray were retrieved from the submitted abstract in NCBI and were subsequently parsed by our in-house python script. The samples used as biological replicates under experimental treatment were identified from the textual annotations.

Microarray normalization was carried out by RMA algorithm in R from Bioconductor package using our customized probeset annotation. To guarantee the statistical power of downstream analysis, we removed the experiments without sufficient biological replicates. Finally, 750 experiments in total were available for ANOVA tests and other downstream analysis. One-way ANOVA test was carried out for each probeset with the experimental conditions as the factor. Two-way ANOVA test was carried out on every APA event with experimental conditions and sub-3′UTR regions as two factors.

### RBP and APA co-expression analysis

A tandem UTR event of *Celf2* was previously reported as a *bona fide* target of NOVA2 by Licatalosi et al. [13]. From the genome-wide NOVA2 binding clusters downloaded from starBase [25], we identified the corresponding NOVA2 binding site on the *Celf2* tandem 3′ UTR region on the genome.

We further identified 101 experiments showing *Nova2* differential expression (One-way ANOVA, *p<0.01*) from 750 microarray experiments. We then found significant differentially expressed APA events (Two-way ANOVA, *p<0.01*) in at least 40 experiments. For each individual experiment, we calculated Pearson correlations between APA ULIs to *Nova2* expression values. For each APA event, we calculated the median value of all correlation coefficients across experiments to characterize the overall correlation between the APA and *Nova2*. *Celf2* APA event appeared among the top list of our result, supporting the validity of our method.

To validate our result from the other perspective, we selected experiments from the microarrays in which *Celf2* APA showed differential expressions (Two-way ANOVA test, *p<0.01*). For each RBP that was differentially expressed (One-way ANOVA, *p<0.01*), we calculated Pearson correlation between its gene expression to ULI of *Celf2* APA. We used the median value of correlation coefficients across experiments to denote the overall correlation strength. We can readily identify *Nova2* in the top list of our result supporting its key regulatory role for *Celf2* APA.

We then applied our method to other significantly differentially expressed APA events. Specifically, the differential expressed APA in a given experiment is defined by Pearson correlation coefficient *r*<−0.4, Two-way ANOVA p<0.01 and mock background corrected Two-way ANOVA p<0.01. We obtained the annotation of RBPs from RBPDB (version 1.2.2) that contained 557 mouse RBP genes [26]. We selected RBPs from within the top 2% positively or negatively correlated genes for each APA. The co-expression network was constructed for all RBP-APA pairs except for the self-correlations between RBP and its own APA events.

### Integration of RBP CLIP-seq data

We downloaded the binding sites and unique CLIP tags derived from CLIP-seq data by starBase (website: http://starbase.sysu.edu.cn/) for Nova2, Tardbp and Ago2 in mouse [25]. For Nova2, we required that the binding sites should have at least four unique CLIP tags to achieve high stringency. For Mbnl1 that lacks the direct information of the binding sites, we conducted the analysis of RBP binding sites by our own CLIP-seq analysis pipeline starting from raw sequencing reads. Specifically, the raw CLIP-seq data of Mbnl1 by Wang et al [27] were downloaded from NCBI Short Reads Archive (accession ID: SRA056973). The raw sequence reads were mapped onto mouse Mm9 genome by Tophat (parameters: –bowtie1 -N 1) and only unique mappings were kept [28]. The script for cluster calling was adopted from our earlier study [29]. Each binding cluster must contain at least four non-redundant reads and 20 total reads.

Next we integrated the gene expression changes for the RBPs from microarray studies with RBP binding site information. We defined RBP differential expression by one-way ANOVA test. The Pearson correlations between RBP expression and ULI of the targeted APA events that contain RBP binding sites were calculated. The APA differential expression was determined by two-way ANOVA (*p<0.01*). The median values of correlation coefficients across all filtered experiments were used to characterize the overall correlation between individual APA event and the RBP in question.

### Methodology

We conducted the Preferred Reporting Items for Systematic Reviews and Meta-Analysis (PRISMA) Statement for our analysis in Checklist S1 [30]. No prior protocol existed for our methods.

## Results

### Construction of mouse poly(A) site database

**Figure 1:**
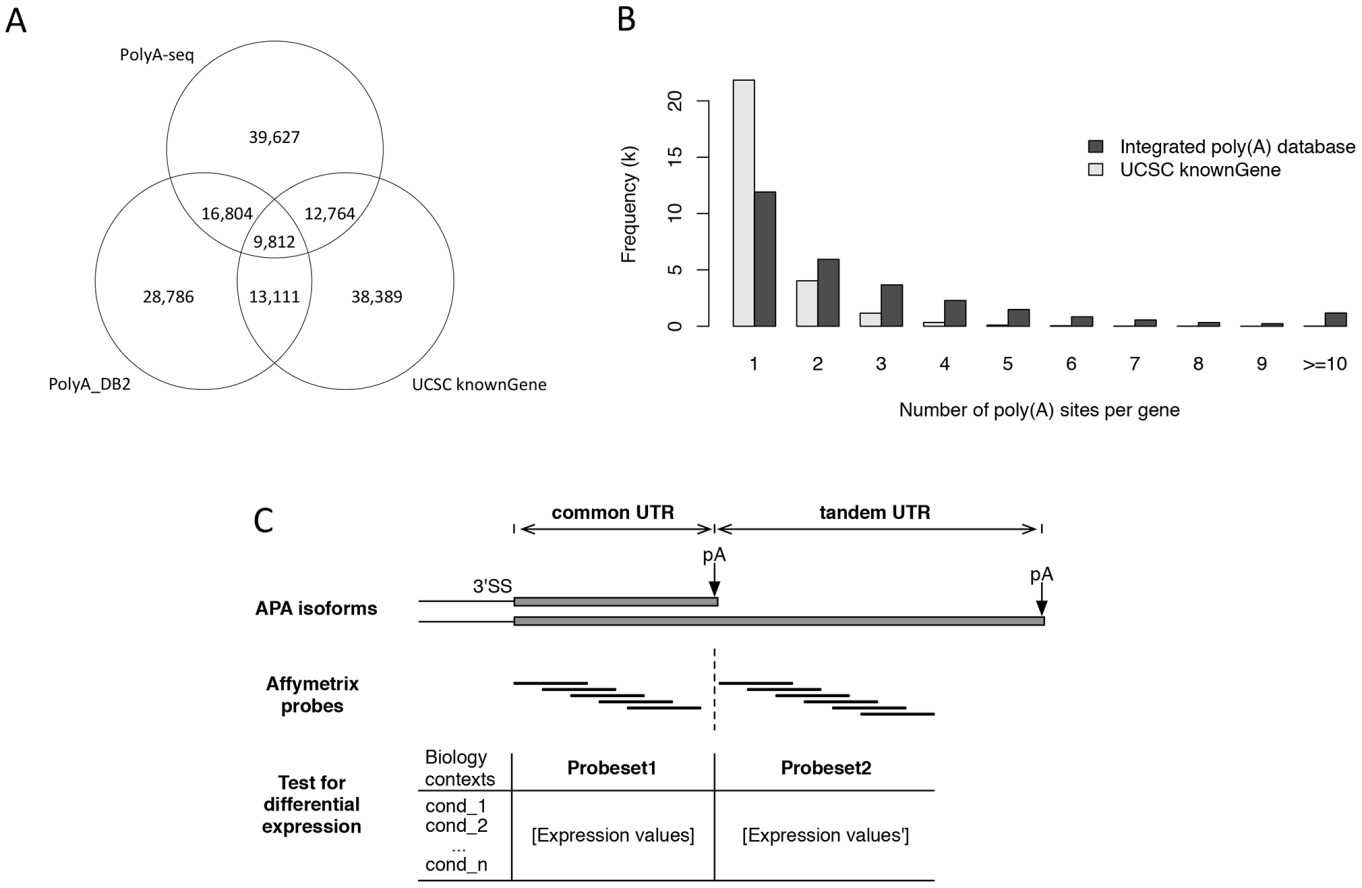
Description of our integrated mouse poly(A) site database. (A). Venn diagram shows the overlaps of three sources of our poly(A) site database including PolyA_DB2, polyA-seq, and 3′ ends of UCSC Known Genes. (B). Histogram shows the distribution of the number of poly(A) site per gene in comparison with UCSC annotation alone. The poly(A) sites based on UCSC Known Gene data alone are colored in grey and our integrated poly(A) database is colored in black. (C). Scheme for probeset customization of microarray probes for APA detection. The expression values of the probesets after quantification and normalization were used for differential APA expression analysis within each experiment.

We first obtained poly(A) site information from both polyA database polyA_DB2 [22] and 3′ ends of all transcripts from UCSC known genes. Next we downloaded a recently published polyA-seq data for five species from UCSC genome browser [5]. A total of 126,028 poly(A) sites were retrieved from mouse polyA-seq results in six tissues. Here we required that the poly(A) site should be detected in at least two tissues to retain 39,710 most reliable poly(A) sites. As a result, we integrated and merged all poly(A) sites from polyA_DB2, 3′ ends from UCSC known genes, and polyA-seq into our own database, resulting 73,935 poly(A) site annotations (Figure 1A). The all-in-one database showed more than half of mouse genes contain at least two poly(A) sites, which suggested the prevalence of APA in the mouse genome. Our annotations of mouse poly(A) sites are much more comprehensive compared with UCSC known gene annotation alone (Figure 1B) (Data in Table S1).

We used the gene annotations from UCSC known genes and extracted their 3′ UTR regions. After incorporating genomic poly(A) sites, the 3′UTR regions can be divided into sub-UTR regions (Data in Table S2), of which expression levels can be measured by corresponding microarray probes (Figure 1C). According to 3′UTR and poly(A) site annotations, we classified three classes of polyadenylation events [31]. Type I event contains only a single poly(A) site. Type II or tandem UTR event contains at least two alternative poly(A) sites on the contiguous 3′ UTR region. Type III or alternative UTR event contains at least two alternative poly(A) sites on different introns or exons.

### Differential APA expression under various biological conditions

To systematically examine the expression of APA events under different biological conditions, we used all existing microarray data in Gene Expression Omnibus (GEO) database (until Sep. 2011) on Affymetrix Mouse4302 microarray platform (Table S3). This type of microarray contains probes mostly designed towards 3′ ends of transcripts and thus can be used to study APA expression [4]. We integrated microarray probe information with our own annotations for poly(A) sites and 3′ UTRs to define our customized probesets. We were able to associate a total of 6,220 type II APA events (tandem 3′UTRs), and 1,859 type III APA events (alternative 3′UTRs) for 4,364 genes with microarray probesets (Materials and Methods, Data in Table S4).

**Figure 2:**
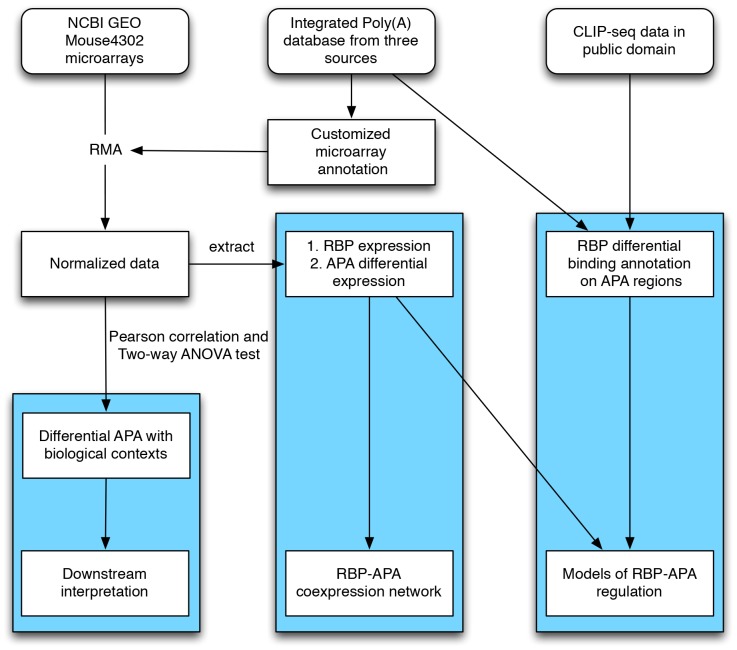
Scheme of the workflow. We generated an integrated poly(A) database and analyzed differential expression of APAs and RBPs in publically available microarrays from GEO aiming to reveal RBP's functions in differential APA regulation. The results of our analysis were highlighted in blue color, which include APA differential expression detection and interpretation in the left panel, RBP-APA co-expression network in the middle panel, and RBP regulation models in APA by combining available CLIP data and microarray gene expression data in the right panel.

The scheme of our meta-analysis pipeline is shown in Figure 2. For a total of 750 microarray experiments, we analyzed differential APA expression using both Pearson's correlation and Two-way ANOVA approaches (Materials and Methods). To remove the experimental bias, we created our customized mock-APA probesets by artificially dividing the probes of the same original Affymetrix probeset into adjacent two probesets. We normalized the correlation coefficients and ANOVA p values of APA probesets by those of mock APA probesets in a given experiment. We characterized the global differential expression of a given APA by integrating the results across 750 microarray experiments. As a result, the top 10 genes with most frequent differential APA events were shown in Table 1. The complete table for differential APA events can be found in Table S5. GO analysis of the genes containing top 120 most significant differential APAs showed that they are enriched in *phosphoprotein* and *nucleotide binding* category. RBPs are enriched in the top list by Fisher's exact test (*p = 0.001, odds ratio = 1.6*). This suggested that differential APA expression of RBPs might be a common phenomenon.

**Table 1:** The genes with most frequent differential APA events.

Genes with APA event	Number of experiments	Most relevant biological conditions
Cd38	83	Tissues (GSE12730), Tumor (GSE14753), Cancer (GSE18534)
Eif4e2	77	Tissues (GSE14395, GSE9441), Development (GSE20954)
Ppp2r5c	67	Tissues (GSE10776, GSE11291, GSE12730), Differentiation (GSE12499)
Cds2	64	Infection (GSE10262), Tissues (GSE11221), Tumor (GSE11990)
Elavl1	61	Tissues (GSE9441, GSE11291)
Araf	58	Tissues (GSE11291), Development (GSE12454), Aging (GSE13120)
Pink1	57	Tumor (GSE11990), Inflammation (GSE13071)
Csnk1d	57	Tissues (GSE10347), Hypoxia (GSE15894)
Mesdc2	57	Tissues (GSE17478, GSE23782)
Wdr33	56	Differentiation (GSE21749), Development (GSE4051)

Number of experiments indicate the number of microarray experiments where the APA event shows differential expression. The biological conditions of the most relevant experiments were listed here.

**Figure 3:**
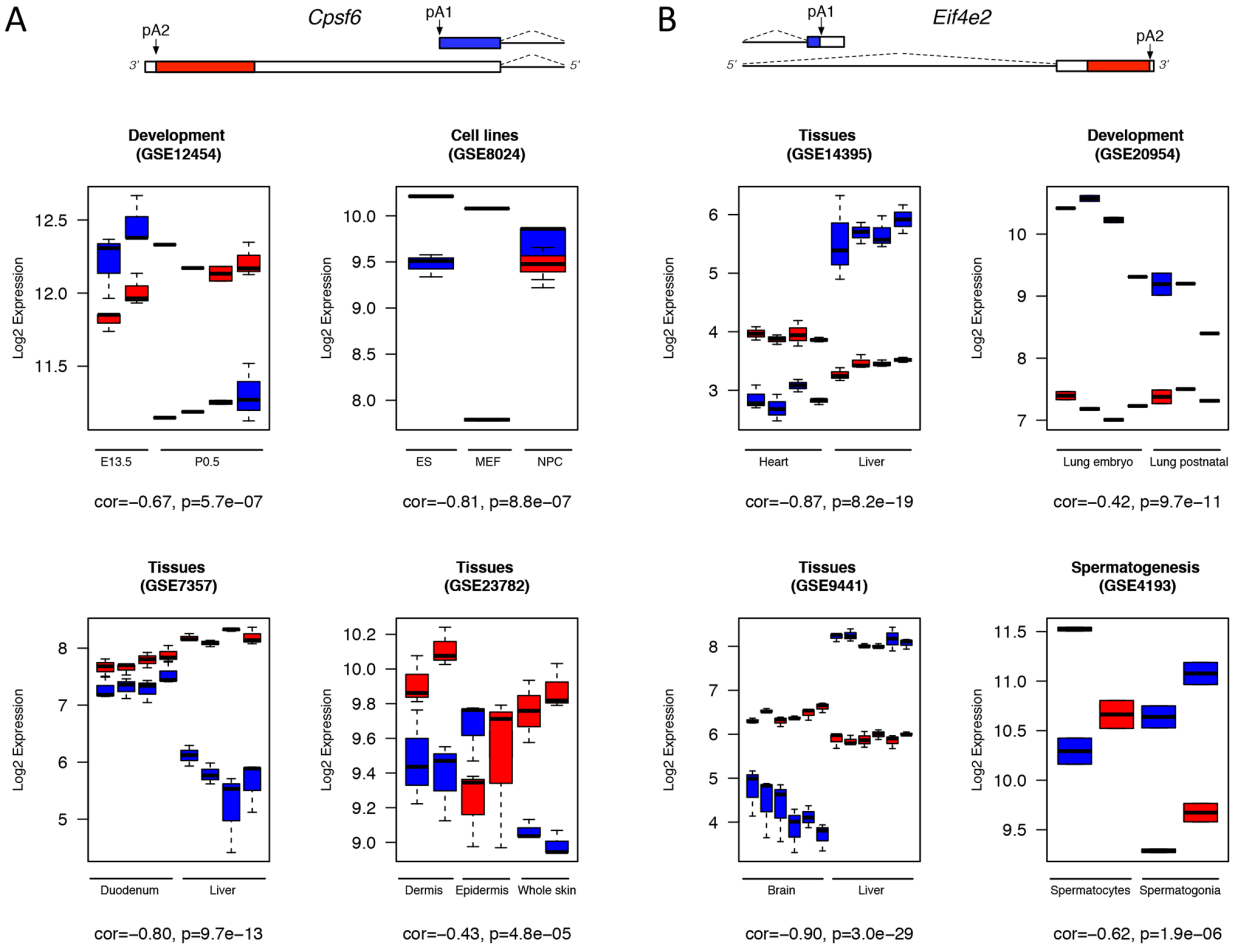
Differential expression of APA for *Cpsf6* and *Eif4e2* in various biological conditions. (A). Boxplots show the differential expression of *Cpsf6* APA isoforms (a tandem UTR event) in different tissues and cell lines, as well as different development stages. (B). Boxplots show the differential expression of *Eif4e2* APA isoforms (an alternative UTR event) in different tissues, development and spermatogenesis. Gene models of the APA isoforms are indicated on top, with microarray probes targeted regions on the 3′ UTRs marked with colors. Blue color represents the common or proximal UTR probeset while red color represents extended or distal UTR probeset. Box-whiskers indicate the expression of probesets across biological replicates within each condition. Y-axis: log2-transformed expression values from microarrays; X-axis: experimental conditions. The descriptions for the biological conditions are listed below each boxplot. *cor*: Pearson correlation coefficient; *p*: two-way ANOVA p value.

For specific APA events, we analyzed the experiment conditions in which they showed significant differential expression (Table 1). We found that the long APA isoform of *Elavl1* was specifically expressed in brain tissues (GEO series: GSE9441, GSE11141, GSE11291). This is consistent with the earlier studies [32]–[34]. Previously we discovered that APA of *Rbm3* showed differential expression in sleep deprivation and cold exposure in mouse [21]. In this study, *Rbm3* is ranked top 22 among the differentially expressed APAs. We identified comprehensive biological conditions leading to *Rbm3* differential APA expression including sleep deprivation (GSE6514, GSE9441), cold shock (GSE20645), hypoxia (GSE14366, GSE15901, GSE9400), and development. From these known cases of differential APA events in *Elavl1* and *Rbm3*, it can be readily seen that our strategy is efficient to identify the biological conditions of differential APA events. We selected two genes with novel differential APAs for illustration in Figure 3. *Eif4e2* ranked top 2 is a cap-binding protein and eIF4E homolog. It showed differential APA expression in different tissues (GSE14395, GSE9441) with short isoform preferred in liver, development (GSE20954) and spermatogenesis (GSE4193) (Figure 3A). *Cpsf6* ranked top 56 is itself a key component of poly(A) machinery. Its differential APA expression suggests its auto-regulation by APA mechanism. We found that the APA isoforms of the *Cpsf6* resulted from tandem UTR were differentially expressed in embryonic development (GSE12454), cell differentiation (GSE8024), and different tissues (GSE7357, GSE23782) (Figure 3B).

### Global shift in APA

**Figure 4:**
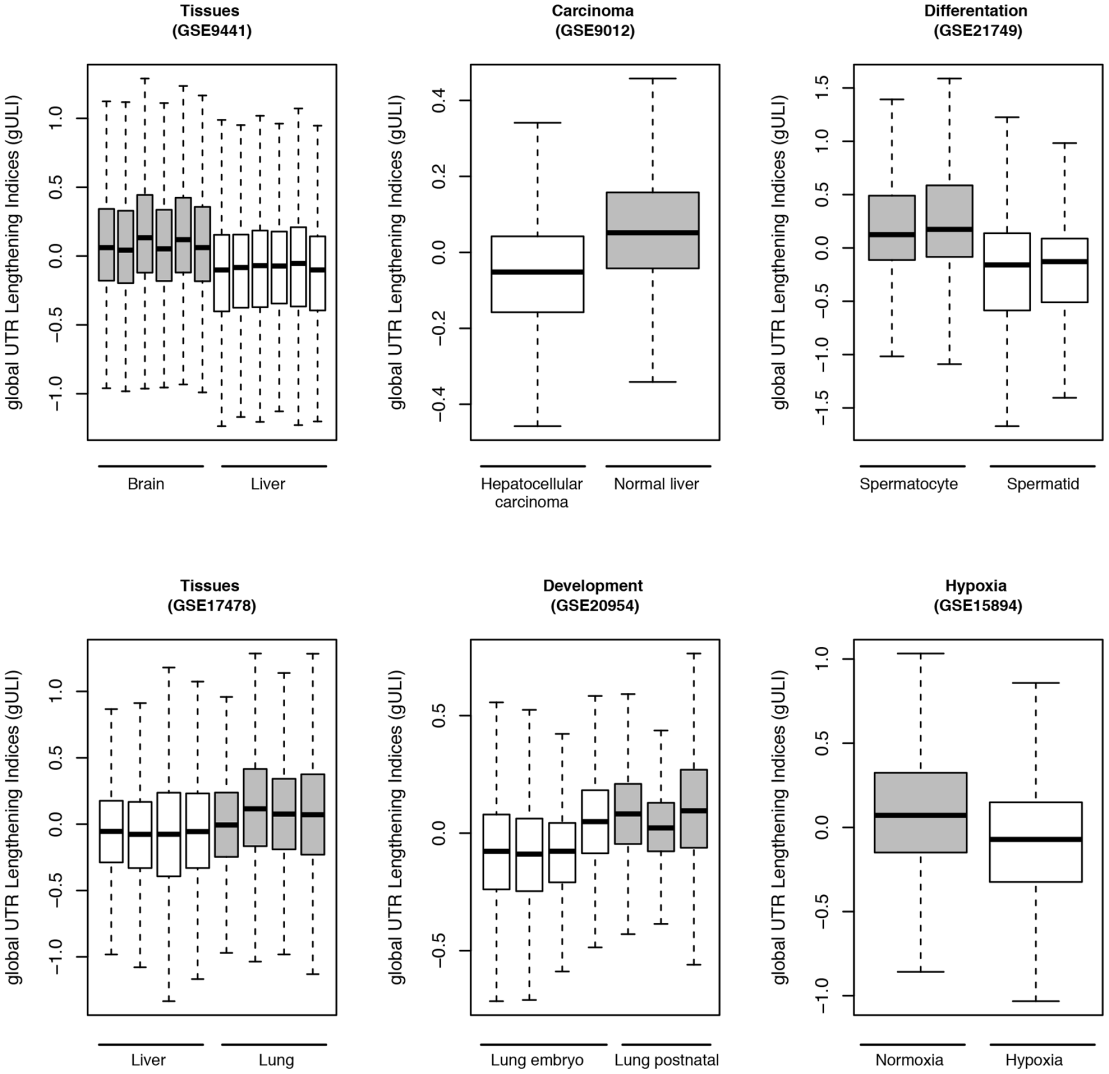
Examples of global shifts in APA. Six experiments were selected from the top list to represent global ULI shifts under different biological conditions. UTR shortening was preferred in certain tissues (GSE9441, GSE17478) as well as in carcinoma (GSE9012), developments (GSE20954), differentiations (GSE21749), and hypoxia (GSE15894). Box-whiskers indicate global UTR lengthening indices (gULI) across biological replicates within each condition. Y-axis: global UTR lengthening index; X-axis: experimental conditions. The descriptions for the biological conditions are listed below each boxplot. Grey color outlines the conditions with lengthening of UTRs while white color for the conditions with shortening of UTRs.

To quantify the global 3′ UTR lengthening or shortening resulted from APA for each experimental treatment, we compared the global distributions of 3′UTR lengthening indices (ULI) across different conditions in a given microarray experiment (Materials and Methods). We observed that some experiments tend to have significantly more differential APAs than the others (Table 2). They encompass diverse experimental conditions containing different tissues, cell lines, developmental stages, cell differentiations, tumors, infections and drug treatments. Brain tissues, especially differentiated neurons, are reported to express extended 3′ UTRs [4], [5], [35], [36]. In our study, we also found that the microarray data conducted in brain tissues tend to express lengthened 3′ UTRs (GEO series: GSE9441, GSE5037, GSE11291). Figure 4 shows one such example that global ULIs in brain tissues are significantly higher than those in liver tissue. Furthermore, we also observed that global 3′ UTR was lengthening in stem cell compare to iPS cells (GSE12499) or differentiated cell types (GSE18669), while 3′UTR shortening events were enriched in tumors (GSE11859, GSE12430, GSE14024, GSE20390), hypoxia (GSE15894, GSE15901), and stress response (GSE8322) as shown in Table 2. Hypothermia in OPC cells mildly resulted global shortening of 3′ UTRs (GSE20645). The complete list can be found in Table S6 (supplementary files).

**Table 2:** The list of biological conditions that most significantly perturb global APA usage.

Experimental categories	GEO experiment series ID	ΔgULI	Biological conditions
Mixed tissues			
	GSE5037	0.34	Spinal cord (−), Brain (+)
	GSE11291	0.32	Heart (−), Neocortex (+)
	GSE9441	0.23	Liver (−), Brain (+)
	GSE17478	0.19	Heart (−), Lung (+)
Cancer or tumor			
	GSE9012	0.1	Hepatocellular carcinoma (−), Normal liver (+)
	GSE8156	0.1	Normal (−), Granulosa cell tumor from mutant mouse (+)
	GSE14024	0.05	CD8 tumor model (−), Tumor suppressed (+)
	GSE20390	0.04	Colon cancer (−), Cancer inhibited (+)
Development			
	GSE3653	0.31	Embryoid bodies 3 days (−), 10 days (+)
	GSE8034	0.29	E14 cortex (−), E18 cortex (+)
	GSE9202	0.28	E18.5 (−), 1 year (+)
	GSE20954	0.18	Lung-embryo (−), Lung-postnatal (+)
Differentiation			
	GSE21749	0.33	Spermatid (−), Spermatocyte (+)
	GSE12499	0.25	iPS cell (−), Neural stem cell (+)
	GSE18669	0.25	T cell (−), Stem cell (+)
	GSE10806	0.22	iPS cell (−), Neural stem cell (+)
	GSE16210	0.2	Activated T reg (−), Naiive T reg (+)
Hypoxia, hypothermia, stress response			
	GSE8322	0.22	ATF6-induced ER Stress in heart (−), Control (+)
	GSE15894	0.14	Hypoxia (−), Normoxia (+)
	GSE15901	0.09	Hypoxia (−), Normoxia (+)
	GSE20645	0.04	OPC 31.5C (−), 37C (+)
Immune response			
	GSE13611	0.28	Control (−), Induced B lymphoid blast crisis (+)
	GSE1871	0.21	LPS-induced lung vascular leak and inflammation (−), Normal (+)

ΔgULI is the maximal shift of global UTR lengthening indices (gULIs).

Several studies have shown that RBPs such as CPSF6 and PABPN1 in human play important roles in the regulation of global shift in APA patterns. To take advantage of large-scale microarray gene expression data in this study, we conducted a co-expression analysis between RBPs and APAs to search for the RBPs that are potentially linked to the regulation of global APA shift. We extracted gene expression for every RBP in a given microarray experiment and calculated their correlations to the global ULI in that experiment. We scored the RBPs by their median correlation coefficients across experiments (Table 3) (Table S7, supplementary files). Our result showed that among RBPs that most correlate with the lengthening of 3′ UTRs are *Tnrc6c|a|b*, *Hnrnpd*, *Rc3h1*, *Helz* and *Cpsf6*. Among them, *Tnrc6c|a|b* are known to silence translation of target mRNAs and *Helz* is a RNA helicase. As a core component of poly(A) machinery, *Cpsf6* enhances the recognition of weak poly(A) sites and was reported to regulate global UTR lengthening in human [37]. Among the RBPs that most correlate with the shortening of 3′ UTRs are *Elavl1*, *Ptbp1*, *Fubp1*, *Cstf2*, and *Ncbp2*. Among them, *Elavl1* is known to destabilize target mRNAs. *Fubp1* is known to activate transcription. *Cstf2* can stimulate mRNA cleavage at polyA sites and *Ncbp2* can promote mRNA 3′-end processing. Taken together, many of the genes that we found using our approach are known to be either directly or indirectly involved in APA regulations.

**Table 3:** The 10 selected RBPs showing the highest positive or negative associations with the global shift in APA.

	Gene symbols with their correlation coefficients
Positively correlated to gULI	Tnrc6c (0.69), Ythdf3 (0.60), Hnrnpd (0.58), Hnrnpa3 (0.58), Phc3 (0.56), Rc3h1 (0.56), Cpsf6 (0.54), Tnrc6b (0.54), Tnrc6a (0.52), Helz (0.50)
Negatively correlated to gULI	Ptbp1 (−0.63), Rbm18 (−0.63), Elavl1 (−0.58), Fubp1 (−0.56), Cstf2 (−0.53), Rrp7a (−0.52), Uhmk1 (−0.52), Ncbp2 (−0.50), Hnrnpab (−0.50), Pno1 (−0.50)

The median correlation coefficients between RBPs and gULIs across experiments are indicated within the parenthesises.

Besides RBPs as the regulatory mechanism of APA, Mercer et al. proposed that uaRNAs resulted from post-transcriptional cleavage could be one of the reasons for differential 3′UTR expression [38]. To check the possibility that uaRNAs could be involved in the differential APA expressions in our study, we adopted 139 genes containing uaRNAs with high fidelity from Mercer et al.'s study. 77 of them are among our APA annotation list. We then examined their differential expression in our results. Among the 368 genes in which APA were differentially expressed in at least 30 experiments, we only found 10 genes containing uaRNAs including *Trip12*, *Fnbp1*, *Tardbp*, *Elavl1*, *Tmpo*, *Ccny*, *Ptch1*, *Cyfip2*, *Cds2* and *Wrb*. Therefore, it seems that uaRNAs play a relatively minor role in differential APA expression considered in our study.

### RBP and APA co-expression network

**Figure 5:**
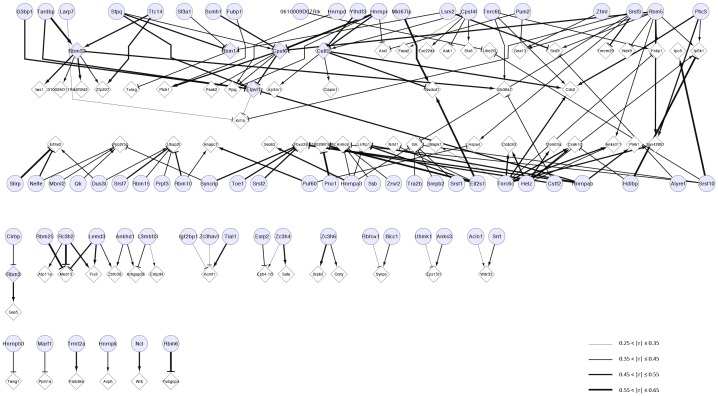
RBP and APA co-expression network. The arrow-headed edge pointing from an RBP indicates that the up-regulation of RBP is correlated with 3′ UTR lengthening of the target gene, while T-headed edge indicates that the up-regulation of RBP is correlated with 3′ UTR shortening. RBP gene is colored in blue and non-RBP gene in white. Gene with APA event is in the diamond shape and gene without APA event is in the round shape. Note that some RBP genes such as *Cpsf6* also contain APA events. The strength of correlation is indicated by thickness of the edges. *|r|*, absolute value of Pearson's correlation coefficient.

For a specific differential APA event, we sought to identify potential RBP regulators by co-expression analysis. We computed the correlations between the ULIs of differential APA events and RBPs. We applied this method to about 50 most significant differential APA events (Materials and Methods). The most correlated RBP-APA pairs were selected to construct a co-expression network shown in Figure 5. Specifically, we found *Cpsf6*, a key component of the poly(A) machinery, as a hub in the network. This finding is consistent with the result that *Cpsf6* expression mainly correlates with global 3′ UTR lengthening. In addition, we identified other RBPs correlated with global APA shifts such as *Tnrc6c*, *Helz* as important hubs in the network.

As a validated case of RBP regulation of differential APA event, Nova2 regulates the tandem UTR lengthening on *Celf2* [13]. In our result, *Nova2* expression was significantly correlated with *Celf2* APA (Materials and Methods, Table S8). Meanwhile, Nova2 CLIP-seq data demonstrated the existence of Nova2 binding sites on *Celf2* APA [13], which further supports that Nova2 is a bona fide regulator of *Celf2* tandem UTR. In the case of *Rbm3* that belongs to type III APA, another cold-induced RNA binding protein *Cirbp* is the most co-expressed RBP with *Rbm3* APA. Other RBPs such as HnRNP family proteins are also among the most co-expressed genes with *Rbm3* APA. This suggests the Cirbp and HnRNP family proteins may play important roles in *Rbm3* APA regulation.

### Incorporation of RBP binding data reveals functional relationship between APA and RBP

**Figure 6:**
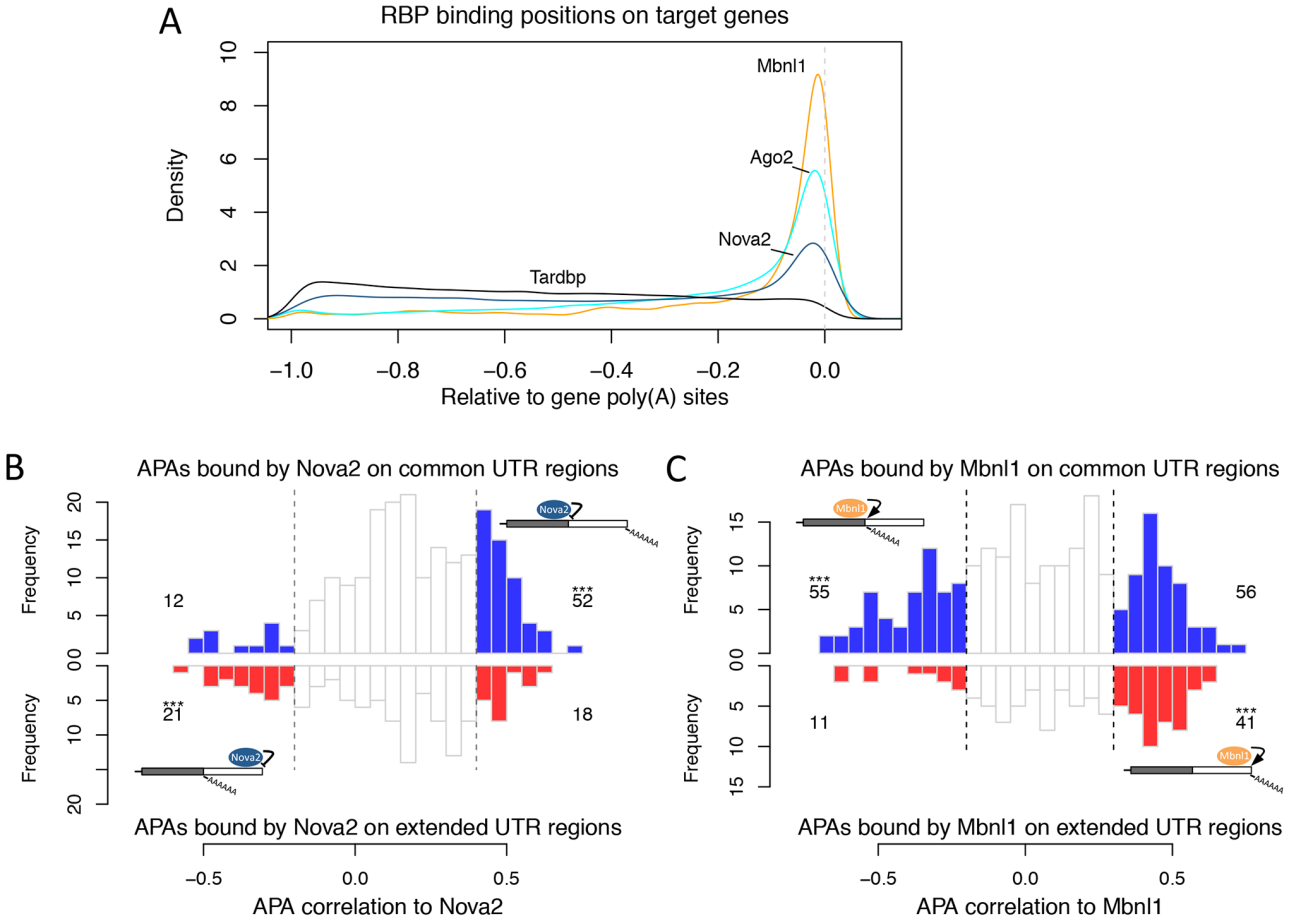
Incorporation of RBP binding data reveals functional relationship between APA and RBP. (A). The distributions of binding sites of Ago2, Tardbp, Nova2, and Mbnl1 on their target genes. On the x-axis, 0 represents the gene 3′ end and −1 represents the 5′ transcription start site of the mRNA. (B). The distributions of Nova2's correlations to its target APA ULIs across microarray expression data when Nova2 binds to common (top) and extended (bottom) 3′UTR regions respectively. We used Pearson's correlation coefficient *r>0.4* and *r<0.2* as the cut-offs of significance. The significant APA events were colored in blue (top) and red (bottom) respectively and their numbers are indicated beside each plot. The statistical significances from Fisher's Exact Tests are indicated for over-represented binding regions (*** p<0.001). (C). Same as in (B) for Mbnl1. Cut-offs of significance are *r>0.4* and *r<0.2*. The models of Nova2 and Mbnl1 regulating APA inferred from our analysis are indicated on the plot. Nova2 supresses while Mbnl1 promotes the usage of the closest poly(A) sites.

As co-expression does not always imply functional relationship, we sought to integrate the RBP-APA co-expression result with RBP physical binding data in order to gain more insights into the mechanism of APA regulation by individual RBPs. At the time of this study, we were able to obtain available CLIP (UV Crosslinking and Immunoprecipitation) sequencing binding data from either PAR-CLIP (photoactivatable ribonucleoside-enhanced CLIP) or CLIP experiments for four RBPs including Ago2, Tardbp, Nova2 from starBase [25] and Mbnl1 from Wang et al [27]. Figure 6 shows the distributions of the binding sites of these RBPs across the mRNA transcript. We found that Ago2 binds predominantly to the 3′ UTR regions. This is consistent with its role in mediating microRNA functions in gene silencing through its interactions with 3′UTRs. In comparison, the binding sites of Tardbp are not enriched in 3′ UTR region but more evenly distributed across the mRNA transcript, consistent with its known function of splicing regulation. The binding sites of Mbnl1 and Nova2 are significantly enriched in the 3′ ends of their target genes (Figure 6A), suggesting their potential roles in APA regulations.

We next investigated the potential regulation of individual APA events by Nova2 and Mbnl1 based on their binding data and microarray expression data. Nova2 binding sites were found closing to 1,388 microarray-detectable poly(A) sites within 200 bp flanking regions, containing 649 type II and 92 type III APA events. Among the type II APA events, Nova2 predominantly binds to the common UTR regions in 370 events as compared to the bindings in the extended regions in 202 events. From 101 microarray experiments where Nova2 was found significantly differentially expressed (One-way ANOVA, *p<0.01*), we calculated the median Pearson correlation coefficients from Nova2 to ULIs of all differentially expressed APA events. We observed that the ULIs of APAs with Nova2 binding to the common UTR regions tend to be more positively correlated with Nova2 expression as compared to APAs with Nova2 binding to the extended UTR regions (Fisher's exact test, p = 0.0004, Figure 6B). This suggests that Nova2 bindings in common UTR regions lead to APA lengthening and Nova2 bindings in extended UTR regions lead to APA shortening. Therefore, Nova2 represses the polyadenylation at the closest poly(A) sites. This is consistent with Licatalosi et al.'s finding that Nova2 blocks the usage of proximal poly(A) sites when binding happens on the common UTR region [13].

We then applied the same analysis on Mbnl1 to examine its role in APA regulation. The binding sites of Mbnl1 were found within 200 bp flanking regions of 1,542 poly(A) sites, containing 571 type II and 82 type III APA events. Among the type II APA events, Mbnl1 also predominantly binds to the common UTR regions in 294 events as compared to the bindings in the extended UTR regions in 140 events. In microarray experiments, we identified 210 experiments in which Mbnl1 gene expression was significantly changed (One-way ANOVA, p<0.005). Similarly, we calculated the median Pearson correlation coefficients from Mbnl1 to ULI of all differentially expressed APA events. Interestingly, ULIs of APAs with Mbnl1 binding to the common UTR regions tend to be more negatively correlated with Mbnl1 expression as compared to the APAs with Mbnl1 binding to the extended UTR regions (Fisher's exact test, p = 0.0006, Figure 6C). This suggests that Mbnl1 has the function in promoting the usage of its closest poly(A) sites, opposite to Nova2 in APA regulation. Based on this analysis, we constructed simple models for Nova2 and Mbnl1 in the regulation of APA as shown in Figure 6B,C. Hence, incorporation of RBP binding data with microarray co-expression analysis can help to reveal specific functional relationship between APA and RBP.

### Online resource for RBP-APA relationships

We have constructed an online website to present our integrated poly(A) site database and result of RBP-APA analysis at (http://www.picb.ac.cn/apa) as a public resource. On this website, user can query and browse poly(A) sites and APA events for genes of interests on the embedded UCSC genome browser. The information such as the 3′UTR regions and microarray probes are also included. For a specific APA event, user can search for the microarray experiments in which it showed differential expression from *APA query* page. From the *RBP query page*, user can query an RBP to retrieve the correlated APA events. The global correlation coefficients between the RBP and the APA events were presented in a result table. Finally, the entire supplementary data in this paper can be downloaded from the *download* page.

## Discussion

Affymetrix microarray has been widely used for gene expression studies and was mainly designed to target genes in 3′ UTR region. It provided us the opportunity to detect subtle 3′ UTR expression changes representing the shift in APA usage among known poly(A) sites. In this study, we re-annotated mouse4302 microarray and developed a computational pipeline to systematically analyze APA differential expression for 1,365 mouse4302 microarray studies from NCBI GEO database. We revealed prevalent differential APA events and their related biological conditions. We found RBPs are enriched in differential APA list. We also observed that global shift in differential APA expression highly depends on biological conditions. While the microarray data can not be used to discover novel poly(A) sites as in high-throughput sequencing, the strength of our approach was the use of large volume of microarray data available in GEO database over a wide range of biological conditions.

Several models have been proposed to describe the mechanisms how RBPs regulate APA expression. The core poly(A) factors, i.e. CPSF and CstF, can directly affect poly(A) site usage. For example, APA in IgM heavy chain in immune system results in two different protein isoforms that are either membrane bound or for excretion. Such APA selection is regulated by CstF-64 [39]. Other RBPs may compete or interact with CPSF or CstF to affect APA [40]. Female specific sex-lethal protein (SXL) competes with CstF in the sex determination of fruit fly [41]. PABPN1 represses polyadenylation by blocking CPSF in human [15]. The expression changes in APA can also be due to the stabilization or degradation of 3′ UTR isoforms differentially regulated by RBPs [42]. RBPs regulate APA expression in a similar way as transcription factors regulate gene expression. Co-expression analysis has been widely used to predict the gene regulation by transcription factors [43], [44]. Meta-analysis on the gene levels has also been applied to the microarray datasets in GEO to reveal functional gene groups [45], [46]. However, our study is the first to draw the links between APA events and RBPs by using co-expression analysis on a large scale. Our meta-analysis result based on the microarrays under different conditions will be valuable to guide future experiments to reveal the detailed regulatory mechanism by RBPs on APAs.

Interestingly, we found that RBPs themselves tend to contain APA events and may be subjected to their own or other RBPs' regulation. It has been observed that RBPs frequently regulate each other to form a complex regulatory network. There are already ample evidences that RBPs play important roles in the regulation of APA. Although other mechanisms such as uaRNAs [38] and epigenetic markers from histone or DNA modifications [47], [48] are also known to affect APA, we focused on RBPs in this study because we can correlate the expression of APA with RBP in public microarray data. In this way, we yielded novel insights into the relationships between APA and RBP. Similar to the study of transcription factors, both expression data and physical binding data are required to understand RBP functions. In the current model, RBPs such as PNBPN1 [15] and CPSF6 [37] cause the lengthening or shortening of 3′UTRs by binding close to the proximal poly(A) sites and thereby block or enhance the recognition of poly(A) sites. To examine this model, we incorporated the CLIP-seq data with microarray data for four selected RBPs. We found that Mbnl1 and Nova2 have clearly distinct roles in the regulation of APA by either promoting or repressing the polyadenylation of closest poly(A) sites. While there are already experimental evidences to support such a mechanism for Nova2 in APA regulation, it is the first time to suggest this novel function of Mbnl1 in APA regulation through our integrative analysis. As more and more RBP binding data such as CLIP-seq data become available, our computational approach can be combined with experimental approaches and deepen our understanding of the complex functions of RBPs in APA regulation.

## Supporting Information

Table S1 **Summarized poly(A) sites database from three Poly(A) data sources for mouse Mm9 genome.**
(XLSX)

Table S2 **Sub-UTR database for mouse Mm9 genome.**
(XLSX)

Table S3 **All GEO microarray experiments used in our meta-analysis.**
(XLSX)

Table S4 **APA events annotated for Affymetrix mouse4302 microarray platform.**
(XLSX)

Table S5 **The list of genes showing the most frequent differential APA events (full list).** For each gene, the experiments in which its APA was differentially expressed are as listed.
(XLSX)

Table S6 **The list of biological conditions that most significantly perturb global APA usage (full list).**
(XLSX)

Table S7 **The list of RBPs showing strong positive or negative association with the global shift in APA (full list).** The median correlation coefficients between RBPs and gULIs across experiments are indicated in an individual column.
(XLSX)

Table S8 **The RBP and APA co-expression network (full network).** Median Pearson correlation coefficients between RBPs and APA targets were indicated.
(XLSX)

Checklist S1 **PRISMA Checklist.**
(DOC)
